# Angiogenesis in the Placenta: The Role of Reactive Oxygen Species Signaling

**DOI:** 10.1155/2015/814543

**Published:** 2015-01-29

**Authors:** Robyn D. Pereira, Nicole E. De Long, Ruijun C. Wang, Fereshteh T. Yazdi, Alison C. Holloway, Sandeep Raha

**Affiliations:** ^1^Department of Pediatrics, McMaster University, Hamilton, ON, Canada L8N 3Z5; ^2^The Graduate Program in Medical Sciences, McMaster University, 1200 Main Street W., Hamilton, ON, Canada L8N 3Z5; ^3^Department of Obstetrics and Gynecology, McMaster University, Hamilton, ON, Canada L8N 3Z5

## Abstract

Proper placental development and function are central to the health of both the mother and the fetus during pregnancy. A critical component of healthy placental function is the proper development of its vascular network. Poor vascularization of the placenta can lead to fetal growth restriction, preeclampsia, and in some cases fetal death. Therefore, understanding the mechanisms by which uterine stressors influence the development of the placental vasculature and contribute to placental dysfunction is of central importance to ensuring a healthy pregnancy. In this review we discuss how oxidative stress observed in maternal smoking, maternal obesity, and preeclampsia has been associated with aberrant angiogenesis and placental dysfunction resulting in adverse pregnancy outcomes. We also highlight that oxidative stress can influence the expression of a number of transcription factors important in mediating angiogenesis. Therefore, understanding how oxidative stress affects redox-sensitive transcription factors within the placenta may elucidate potential therapeutic targets for correcting abnormal placental angiogenesis and function.

## 1. Introduction

The placenta is located at the maternal-fetal interface and modulates the* in utero* environment to promote optimal fetal development. The dense networks of blood vessels within the placenta are responsible for exchanging respiratory gases, nutrients, and wastes between the mother and fetus throughout pregnancy, which is essential for proper fetal growth [1]. Throughout gestation the vasculature of the placenta is continually evolving to accommodate the mounting demands of the fetus and can be directly influenced by a number of exogenous factors such as maternal diet, smoking, and medication use [2–5]. Furthermore, conditions which subject the placenta to stress, such as increased dietary fats and exposure to the chemicals in cigarette smoke, can also result in altered levels of immune and growth factors which may impact the proper development of placental vasculature [6–8].

The establishment of proper placental function first requires successful implantation of the fertilized oocyte followed by the coordinated invasion of trophoblast cells, from the trophectoderm layer of the blastocyst into the maternal decidua. Following invasion, trophoblasts remodel the maternal spiral arteries to promote expansion of the placenta's vascular circuitry, which is central to improving uterine and umbilical blood flow. This facilitates efficient exchange of nutrients and thereby permits exponential fetal growth and development [9–11].

Abnormal development of the placental vasculature leads to placental insufficiency, which can result in a decrease in the exchange of nutrients and wastes between maternal and fetal circulations. Such changes can manifest in adverse uterine conditions leading to various pregnancy complications for both the mother and the fetus including gestational hypertension [12], intrauterine growth restriction [13, 14], preeclampsia [15, 16], stillbirth [17, 18], preterm delivery [19], or miscarriage [20]. Therefore, the proper establishment of blood vessels within the placenta is central to fetal growth and survival and may serve as a therapeutic target for mitigating clinical conditions that are associated with altered placental vasculature.

Appropriate development of the placental vascular network requires vasculogenesis, angiogenesis, and trophoblast mediated arterial remodeling [21]. Vasculogenesis is the development of blood vessels* de novo* from pluripotent mesenchymal stem cells, occurring between 18 and 35 days after conception in humans. Angiogenesis is the creation of new blood vessel networks by branching and elongating previously existing vessels to make new connections. Angiogenesis occurs during most of the pregnancy, beginning 21 days after conception and continuing throughout human gestation [16, 22]. It is important to recognize that vascular development is mediated not only by the vascular endothelial cells, but also by the invading trophoblast cells. The extravillous trophoblasts contribute to the development of the placental vasculature by secreting angiogenic factors [23, 24] and invading the maternal decidua to remodel maternal spiral arteries creating a “low resistance, high capacitance vessel,” increasing the exchange between maternal and fetal circulations [16]. All of these functional changes require the coordinated actions of various signaling molecules to regulate the expression of genes that govern placental vasculature development [25].

In order to elucidate the mechanistic signaling pathways involved in the development of the placental vasculature, researchers have sought to identify the maternal and fetal exposures that are linked to placental dysfunction. For instance, maternal smoking, obesity, and preeclampsia are three conditions that are linked to altered placental angiogenesis. Examining placental vascular development in these conditions may provide us with greater insights into factors that are important for proper placental blood vessel development.

## 2. ROS Signaling in Placenta Angiogenesis

Reactive oxygen species (ROS) are hyperreactive molecules resulting from the reduction of molecular oxygen. Some of the most commonly known species are superoxide (O_2_^−•^), hydroxide (OH^−•^), and hydrogen peroxide (H_2_O_2_) [26]. These ROS are primarily formed from mitochondrial oxidative phosphorylation, where electrons are transferred across respiratory chain enzymes and leak onto molecular oxygen. At physiological levels, ROS are involved in cellular signaling pathways important for proper development and cellular function. However, excess ROS can cause cellular damage and impact tissue function as a result of lipid peroxidation, protein and amino acid modifications, and DNA oxidation. Antioxidants are molecules and enzymes capable of reducing the consequences of these ROS and/or mitigating their damaging effects [27, 28]. Typically, there is a dynamic balance between the generation of ROS and the actions of antioxidants. This balance is critical for maintaining ROS at optimal levels for signaling of various cellular processes, while avoiding a state of damaging oxidative stress [29].

**Figure 1:**
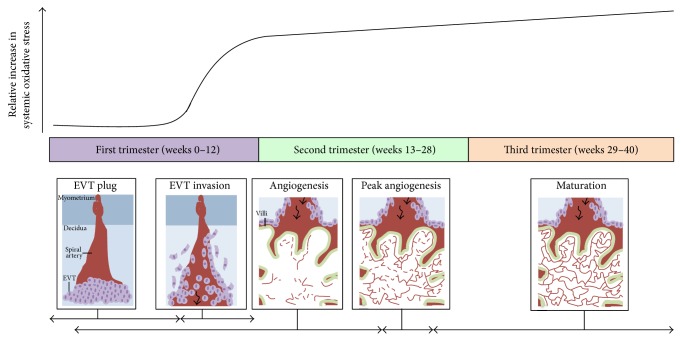
Oxidative stress throughout human pregnancy and its relation to placental angiogenesis. At the beginning of the first trimester of pregnancy, there are low levels of systemic oxidative stress and no blood flow into the placenta because extravillous trophoblasts (EVTs) (depicted as light purple circles) plug the maternal spiral arteries (depicted in red) in the decidua as shown in the first panel. Between 8 and 12 weeks of gestation, the EVT plug dissipates and the EVTs invade maternal spiral arteries to allow blood to enter the placenta (black arrow), as illustrated in the second panel. This coincides with a sharp increase in maternal oxidative stress. Furthermore, the state of oxidative stress increases with gestational age as depicted by the black curve. The first signs of placental angiogenesis occur at 3 weeks of gestation. However from about 12 weeks onwards, blood vessels (red lines) protrude towards the trophoblastic layers of the villi (outlined in green), where blood exchange between maternal and fetal circulation is optimal (shown in panel three). From about 9–23 weeks of gestation, there is an expansion of the fetal capillary bed by branching and nonbranching angiogenesis (dashed red lines in angiogenesis panel). From 23-24 weeks of gestation, the greatest changes in blood vessel development and villous composition are observed (peak angiogenesis panel) [22, 34]. Angiogenesis continues until term with the maturation of blood vessels and development of a more complex vascular network to facilitate exponential fetal growth (last panel). The horizontal black arrows indicate the approximate time each process depicted in the panels occurs. Note: this graph has been constructed by interpretations of multiple studies reporting findings of systemic oxidative stress markers present in women during normal pregnancy [35–40] as studies on placental/uterine oxidative stress are limited [32].

The effects of altered placental oxygenation, with increasing gestational age, on placental development have been nicely reviewed by Burton [30]. Fluctuating oxygen conditions can contribute to increased ROS production where they can act as signaling molecules. This may be particularly pertinent to tissues which have a high-energy demand or those which contain large amounts of mitochondria, such as the placenta [31], brain, heart, and skeletal muscle. Moreover, ROS production changes over the course of pregnancy, underscoring the importance of oxidative signaling in the placenta. In the early stages of human pregnancy, the establishment of placental circulation is associated with a dramatic increase in the oxygen level within the placenta [32], resulting in increased ROS production and oxidative stress. As pregnancy progresses and the metabolic demands of the fetus rise, there is an increase in both placental mitochondria mass and mitochondrial electron chain enzyme activity. This contributes to elevated ROS production and increased oxidative stress [33]. Moreover, there is an increase in systemic oxidative damage during the course of human pregnancy which corresponds to extravillous trophoblast invasion and the development of the placental vasculature (Figure 1). Therefore, it is possible that ROS signaling during human pregnancy is important for central processes such as placental vasculature development.

There are a number of maternal conditions such as smoking, obesity, and preeclampsia that can disturb the profile of ROS production that occurs during healthy pregnancies. Moreover, because maternal smoking, maternal obesity, and preeclampsia can also cause placental dysfunction and pregnancy complications, it is important to elucidate the contribution of excessive ROS production and increased oxidative stress to proper placental function and pregnancy outcomes. Here we explore the existing associations between altered placental angiogenesis and oxidative stress in conditions of maternal smoking, maternal obesity, and preeclampsia.

## 3. Smoking and Angiogenesis

15–20% of women smoke during pregnancy despite maternal smoking being a significant risk factor for a number of adverse pregnancy outcomes [41]. Maternal smoking is associated with an increased risk of fetal growth restriction, low birth weight, and perinatal mortality [41–43]. Such changes are often linked to abnormal placental development and specifically to aberrant placental vascularization. Indeed, an increase in capillary/villous tree branching and vascular density within the placental terminal capillary convolutes has been observed in women who smoke. In such cases, poor development of the placental vasculature was inferred by an increased umbilical artery Doppler resistance index which is indicative of abnormal vascular tree formation [44]. Exposure of placental explants to cigarette smoke extracts has also been shown to shift the balance between proangiogenic (promoted by placental growth factor, PlGF) and antiangiogenic factors (fms-like tyrosine kinase-1, sFlt-1) [3]. Moreover, a number of animal studies have demonstrated that maternal chronic exposure to carbon monoxide, a major combustion product produced by cigarette smoke, increases uterine blood flow and uteroplacental vascular growth by shifting the placenta to a more proangiogenic state [45]. However, some controversy still exists with respect to the proangiogenic effects of smoking during pregnancy; several studies report a reduction in the number of placental capillaries among smokers [46, 47]. In fact, the addictive component of cigarette smoke (nicotine) can inhibit trophoblast migration, a key process initiating trophoblast invasion and spiral artery remodeling, within human placental explants [48]. Recently it has been shown that nicotine inhibits trophoblast interstitial invasion, downregulates transcription factors required for trophoblast differentiation, and impairs placental vascularization [49]. Therefore, maternal smoking clearly influences both placental development and function; however there is still considerable uncertainty regarding the mechanism(s) underlying these effects.

Smoking is generally considered to be an oxidative insult and as a result it has been suggested that smoking causes abnormal placental vascular development via oxidative stress mediated pathways. Although this has not been shown unequivocally, recent studies support that maternal smoking results in changes in genes important for the management of oxidative stress [50], which may contribute to adaptive changes in the state of oxidative stress within the placenta. For example, smoking throughout pregnancy has been associated with an increased expression of antioxidants, such as heme-oxygenase within the basal plate of term placenta as well as in HTR8/SVneo trophoblast cells exposed to cigarette smoke extract [51]. Sidle and colleagues suggested that the increased expression of heme-oxygenase within the placenta may be facilitating trophoblast invasion of the spiral arteries and thus decreasing the placental oxidative damage incurred from smoking. However, increased oxidative damage to lipids and DNA is observed within term placenta of women who smoked during pregnancy [52]. Additionally, the total antioxidant capacity was significantly lower in the placentae of active smokers, while the total oxidant status and oxidative stress index was significantly increased in both the placentae from active and the passive smokers compared to placenta from nonsmokers during pregnancy [53]. Taken together, the current body of evidence suggests that oxidative stress dependent mechanisms play an important role mediating the effects of cigarette smoke on the placenta, potentially by influencing the development of the vascular network.

## 4. Maternal Obesity and Angiogenesis

Maternal obesity during pregnancy also results in adverse maternal and fetal outcomes including gestational diabetes, preeclampsia, macrosomia, preterm delivery, and stillbirth [6, 54–56]. Many of these outcomes have also been associated with alterations in the placental vasculature. Indeed, maternal obesity has been clinically linked with placental abruption and infarction [57] along with abnormal placental spiral artery modification, which may result from inadequate trophoblast invasion [17, 58]. The importance of proper trophoblast invasion in dictating functional development of the placental vasculature is well supported [59, 60]. In fact, our group has demonstrated that the altered progression of trophoblast invasion is correlated to incomplete spiral artery remodeling in rodents exposed to a life-long high-fat diet [7]. Other animal models of obesity during pregnancy have also reported impaired placenta vascular development and increased capillary density [61] as well as significant reductions in uterine blood flow [62]. Additionally, a study done with obese ewes found that the fetal component of the placentomes displayed larger arteriole diameter in early to midgestation, with a decreased gene expression of angiogenic factors from mid to late gestation [63]. Therefore, maternal obesity causes adverse development of the placental architecture, which results in poor nutrient and waste exchange, in turn compromising fetal growth and survival [6, 62, 64]. Mechanistically, a higher body mass index during pregnancy is associated with altered levels of maternal serum angiogenic markers (low levels of sFlt-1 and altered levels of PlGF after first trimester and differential patterns of change near term) [65]. Abnormalities in the distribution of proangiogenic vascular endothelial growth factor (VEGF) and its receptors in the placentae of obese women have also been noted, with a particular mention of a predominance of nonbranching angiogenesis [66].

Maternal obesity has also been linked with placental oxidative stress. First trimester placenta from obese pregnancies has been shown to have a 31% increase in total oxidized protein content (a marker of oxidative damage) compared to placenta of nonobese pregnant women [67]. Term placentae from obese women also have altered redox balance as indicated by increased lipid peroxidation (malondialdehyde measurement) and activity of antioxidant enzymes such as the superoxide dismutases, catalase, and glutathione peroxidase, compared to control placenta [68]. However, the role of oxidative stress in the placentae of obese individuals is still not clear since some research suggests that there is a decrease in total antioxidant capacity and regulators of angiogenesis within term placentae of obese pregnancies compared to nonobese placentae [2]. In addition to oxidative stress, the work of Myatt and colleagues has also raised the possibility of nitrative stress being a route to vascular dysfunction in the placentae of obese women [69, 70]. Elucidation of the transcriptional and posttranscriptional processes that contribute to obesity-mediated changes in placentae will advance our understanding of the mechanisms linking oxidative stress to functional changes in the placenta, such as those involved in placental angiogenesis.

## 5. Preeclampsia and Angiogenesis

Preeclampsia can lead to intrauterine growth restriction, preterm delivery, and stillbirth [71]. There are a number of good reviews that discuss the connection between altered placental vascular development and preeclampsia [72–74], many of which highlight the role of elevated oxidative stress in causing reduced trophoblast invasion as a mechanism underlying the development of preeclampsia [33, 70, 75]. A recent proteomics evaluation of preeclamptic placentae demonstrated a reduction in the levels of several mRNAs associated with mitochondrial respiratory chain function [76]. Since mitochondria are intimately associated with oxidative stress and signaling [29, 77], understanding their role in dictating placental function/dysfunction may be important to elucidating disease pathogenesis [78].

**Figure 2:**
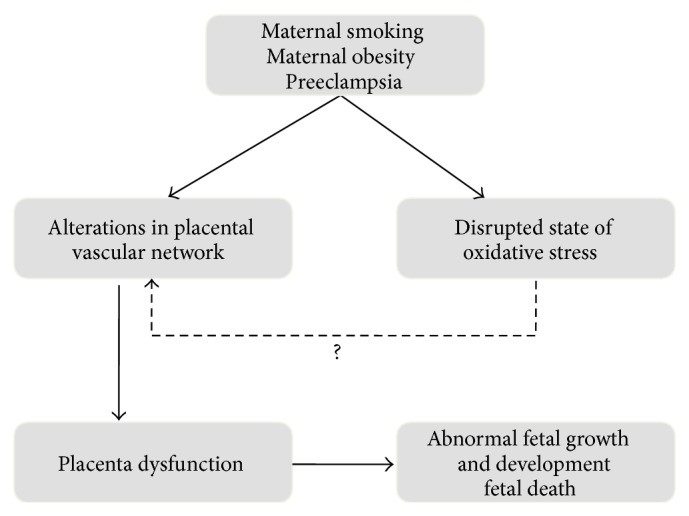
Summary of proposed mechanisms linking maternal smoking, maternal obesity, and preeclampsia with pregnancy complications and adverse fetal outcomes. It is well documented that both the placental vasculature and the state of oxidative stress are altered in pregnancies complicated by maternal smoking, maternal obesity, and preeclampsia (see text). These alterations in the placental vascular network are known to contribute to placental dysfunction and adverse pregnancy outcomes as well as abnormal fetal growth, development, and even death. We hypothesize that an increase in oxidative stress contributes to aberrant signaling in placenta, resulting in changes in processes essential for placental vascular development. This could be a potential mechanism leading to the adverse pregnancy outcomes observed in maternal smoking, maternal obesity, and preeclampsia. The solid arrows represent well documented findings, while the dashed arrow identifies the knowledge gap where more research needs to be done, to clarify the role of oxidative stress in placental angiogenesis.

While oxidative stress in the placenta is associated with adverse pregnancy outcomes, the mechanistic connection between the role of ROS and altered vascular development is not clearly defined (Figure 2). Therefore, the elucidation of how oxidative stress/signaling facilitates placental dysfunction as a consequence of maternal smoking, obesity, and preeclampsia will contribute to a better understanding of the specific cellular pathways linking these uterine stressors to placental dysfunction.

## 6. Putative Role of ROS Activated Transcription Factors in the Placenta

While oxidative stress has been proposed as a contributory pathway for many instances of placental dysfunction, the association of specific signaling pathways with ROS still remains unclear. Therefore, in this section, we describe the roles of six transcription factors which may link oxidative stress with trophoblast invasion and vascular development in the placenta [79]. Specifically we address the roles of E26 transformation specific oncogene homolog 1 (Ets-1); Krüppel-like factor 8 (KLF8); nuclear factor kappa-light-chain-enhancer of activated B (NF-*κ*B); NF-E2-related factor 2 (Nrf2); specificity protein 1 (Sp1) and specificity protein 3 (Sp3); and signal transducer and activator of transcription 3 (STAT-3).

### 6.1. Ets-1

Transcription factor Ets-1 is upregulated by hypoxia and ROS and regulates angiogenesis and invasion [80, 81], processes central to the normal development and function of the placenta. For instance, in bovine aortic endothelial cells it has been demonstrated that Ets-1 mRNA levels are increased by hypoxic conditions as well as elevated levels of cellular ROS [82]. More specifically, in endothelial cells and ovarian carcinoma cells, H_2_O_2_ has been shown to regulate Ets-1 through the hypoxia response element (HRE) and the antioxidant response element (ARE) [80]. In general, the Ets family has been linked with a number of other cellular processes such as apoptosis [83] and cellular differentiation [84]. Furthermore, other transcription factors, such as Nrf2, also play a role in the upregulation of Ets-1, by forming a complex with Ets-1 that associates with the ARE [80]. Under hypoxic conditions, other transcription factors such as hypoxia-inducible factor (HIF) can also trigger the increased expression of Ets-1 [81].

While conditions that increase free radical signaling can regulate the expression Ets-1, Ets-1 itself upregulates VEGF, a key protein in angiogenesis [85]. Additionally, Ets-1 can also interact with other transcription factors known to be important for cellular responses to oxidative stress and hypoxia, such as HIF-2*α*, to regulate the expression of VEGF receptor 2 (VEGFR-2) [86]. Furthermore, proangiogenic factors, such as VEGF, also induce Ets-1 expression in human umbilical vein endothelial cells which then goes on to bind to the promoter region of angiopoietin-2, upregulates the protein, and destabilizes vessels for angiogenesis [87]. Clearly Ets-1 has important roles in regulating the angiogenic response in a variety of cell types; however its role in the placenta is not clearly understood. We do know that Ets-1 expression in normal human placenta correlates with trophoblast invasion and has been observed to peak during the first trimester [88]. A number of investigators have suggested that, based on its correlation with increased invasion, Ets-1 may serve to increase the expression of matrix metalloproteinase 9 (MMP-9) and urokinase-type plasminogen activator (uPA), which are important players in trophoblast invasion [89].

Importantly, the regulation of Ets-1 dependent signaling pathways has been linked to the dysregulation of genes in smoking, obesity, and preeclampsia. While the role of Ets-1 in placenta is not well characterized, the observations outlined below argue for the importance of examining the role of Ets-1 dependent signaling pathways in placental vascular development and function. Firstly, the expression of MMP-1 is regulated in human epithelial cells as consequence of exposure to cigarette smoke [90] through an Ets-1 dependent pathway. Secondly, the addition of serum from obese humans increases MMP-3 and Ets-1 expression in human aortic endothelial cells [91]; this increase may be triggered by serum VEGF. The current evidence suggests that Ets-1 is specifically associated with proteins important to trophoblast invasion (such as the MMPs) and placental angiogenesis (such as VEGF), parameters that are frequently altered in smokers and obese individuals. Along with evidence that hypoxia and oxidative stress can also increase Ets-1 expression, it suggests that this transcription factor may play an important role in pregnancies complicated by oxidative stress.

### 6.2. KLF8

KLF8 is important in facilitating cellular differentiation [92, 93] as well as angiogenesis [94]. KLF8 has been extensively investigated within the context of different cancers, including hepatocellular carcinoma and breast cancer, where its activity has been found to induce invasion and metastasis [93, 95]. It has been suggested that KLF8 also plays a role in the activation of MMP-9 in breast cancer, and this may be an important signaling mechanism underlying invasion and metastasis [95]. Furthermore, the work of Yang et al. (2014) suggests that hypoxia-reoxygenation (H/R) also serves to reduce the expression and nuclear colocalization of KLF8 resulting in a downregulation of MMP-9, which ultimately inhibits trophoblast invasion [96]. Since H/R leads to the production of ROS which can modulate protein kinase C (PKC) [97] and KLF8 has a binding site for PKC, it is possible that the altered interaction between KLF8 and PKC may contribute to the reduction of nuclear KLF8. This hypothesis is further supported by research showing that matrix degradation by MMP-9 is PKC dependent; thus a decrease in PKC activity would also reduce the matrix degrading activity of trophoblasts [96]. While we do not know the exact role of KLF8 in the placenta, the existing evidence suggests that it may be regulated in conditions that affect trophoblast invasion, especially as a consequence of oxidative stress. In support of this hypothesis, decreased KLF8 expression has been associated with reduced MMP-9 mRNA and protein expression in trophoblasts from preeclamptic placentae. Furthermore, HTR8 human trophoblast cells subjected to H/R injury, which mimics changes in oxygen tension observed following the initiation of trophoblast invasion, also downregulate KLF8 and MMP-9 expression [96].

In addition to its effects in preeclampsia, KFL8 also plays a role in 3T3-L1 adipocyte differentiation by acting as an upstream regulator of peroxisome proliferator-activated receptor gamma (PPAR*γ*) [92]. If KFL8 also regulates PPAR*γ* in the placenta, a key factor in placental angiogenesis [98], it is plausible that under obesogenic conditions KLF8 may also have an important role in altering the development of the placenta vasculature. Therefore, KLF8 is another transcription factor of interest in linking oxidative stress with altered placental development and angiogenesis.

### 6.3. NF-*κ*B

Perhaps the most recognized transcription factor associated with ROS signaling is NF-*κ*B. Increases in cellular ROS production can result in an increase in the expression of NF-*κ*B, leading to the upregulation of factors involved in angiogenesis [99, 100]. Furthermore, the addition of H_2_O_2_ to endothelial cells has been shown to result in the increased expression of VEGFR-2 mRNA via ROS and NF-*κ*B dependent pathways. In addition to NF-*κ*B inducing angiogenic factors and the expression of their receptors, it can also be activated by angiogenic factors such as VEGF [101]. Such a relationship can potentially amplify the angiogenic response triggered by cellular oxidative stress.

NF-*κ*B can also influence angiogenesis by regulating the expression of cytokines such as interleukin-6 (IL-6) and interleukin-8 (IL-8), as shown in cancer cell models [102, 103]. The role of NF-*κ*B may be to link the detection of ROS to regulation of cytokine expression. For example, H_2_O_2_ can stimulate the cellular levels of NF-*κ*B and also increase DNA binding activity of NF-*κ*B resulting in an increase in the production of IL-8 and the formation of tube-like structures [99]. Cytokines can regulate angiogenesis by directly acting on cell growth and differentiation and indirectly by inducing the release of secondary cytokines that influence the expression of angiogenic factors or receptors (reviewed in [104]).

Preeclamptic placentae express increased levels of NF-*κ*B; in some cases the increase is reported to be as much as 10-fold [105]. More recent evidence suggests that other conditions leading to placental dysfunction, such as increased weight gain [106] and exposure to a high fat diet [2], may also result in increased expression of NF-*κ*B. Such conditions are linked to increased oxidative stress and placental dysfunction [7, 69], providing justification for further examining the role of NF-*κ*B in connecting placental oxidative stress to angiogenesis.

External stressors such as diet and cigarette smoke are known to affect changes in NF-*κ*B expression in a variety of tissues. For instance, exposure of human airway epithelial cells to cigarette smoke increases the expression of NF-*κ*B within 2 hrs [90]. It has also been suggested that aldehydes, present in cigarette smoke, may interfere with NF-*κ*B binding of its target promoters [107]. Taken together these data suggest that NF-*κ*B may be an important link between oxidative stress, smoking, and angiogenesis. There is also strong evidence that NF-*κ*B is upregulated in the placenta of obese ewes [108] as well as obese women [2]. However, whether this increase is strictly the consequence of increased oxidative stress in these placentae is not clear.

In summary, ROS and oxidative stress can regulate the expression of NF-*κ*B in a variety of tissues leading to changes in angiogenesis. Therefore, NF-*κ*B may be having the same regulatory effect within the placenta and be a key transcription factor in regulating ROS-induced placental angiogenesis.

### 6.4. Nrf2

Nrf2 is a transcription factor that is involved in the regulation of nonmitochondrial antioxidant defense response [109], as well as mitochondrial proteins related to the management of ROS production [110]. Nrf-2 and its partner Kelch-Like ECH-Associated Protein 1 (Keap-1) have been linked to the oxidative stress response in tissues such as adipocytes [111], as a consequence of obesity, and in peripheral blood mononuclear cells, as a consequence of smoking [112]. Furthermore, increased expression of Nrf2 in the placenta is associated with preeclampsia and fetal growth restriction [113]. In such cases, trophoblast invasion is usually reduced and this is thought to be the result of increased cellular oxidative stress [114]. However, the link between Nrf2 and angiogenesis in the placenta is less clear.

Nrf2 is also associated with proangiogenic potential in endothelial cells [115] and may be triggered in response to reduced oxygen conditions. Nrf2 is thought to have its proangiogenic effects by modulating known regulators of angiogenesis, such as VEGF, in response to oxidative stress [116]. This has been demonstrated in extravillous trophoblasts where the placentae from women with early onset preeclampsia and intrauterine growth restriction exhibited increased levels of Nrf2 expression in association with decreased expression of VEGF and elevated levels of 4-hydroxynonenal (a marker of lipid oxidative damage) [113]. Since VEGF is also associated with the activation of Nrf2, perhaps the early decrease in VEGF may lead to insufficient activation of Nrf2 and this may play a part in modulating trophoblast invasion [117]. Clearly our understanding of the role of Nrf2 in linking trophoblast oxidative stress to placental angiogenesis is incomplete and requires further investigation. 

### 6.5. Sp1 and Sp3

Sp1 and Sp3 are zinc finger proteins that are ubiquitously expressed in most mammalian cell types and are associated with enhanced gene promoter activity [118] by binding to GC boxes [119]. While there is little direct evidence for the role of Sp1 and Sp3 in placental angiogenesis, both of these transcription factors have been associated with the regulation of VEGFR-2 in pancreatic tumors [120]. Furthermore, Sp3 has been linked to the regulation of VEGF through a mechanism involving the posttranslational phosphorylation of a serine residue by extracellular related kinases [121]. In addition Sp1 has also been shown to modulate another transcription factor, PPAR-*γ* [122], which is known to be involved in placental angiogenesis [98]. Both Sp1 and Sp3 are also thought to be involved in the regulation of angiogenesis by cytokines such as IL-6 [123]. Such mechanistic pathways may be important in cases of altered placental angiogenesis due to obesity since cytokine balance is often affected as a consequence of overnutrition. Therefore, Sp1 and Sp3 may be important transcription factors in regulating placental angiogenesis through their regulation of VEGF, VEGFR-2, and PPAR-*γ*, as well as cytokines.

Oxidative stress, generated by the addition of exogenous H_2_O_2_, was also found to increase VEGF-A expression via Sp1 and Sp3 dependent pathways, by binding to two GC boxes in the VEGF-A promoter region [124]. In addition, Sp1 has been linked to the regulation of tissue inhibitors of metalloproteinase-2 (TIMP-2) [125]. TIMP proteins are important in regulating MMP activities that are central to trophoblast invasion and spiral artery remodeling. Furthermore, cigarette smoke has been shown to induce MMP-1 expression, an enzyme that is important in angiogenesis, through Sp1 dependent pathways [90]. Clearly, Sp1 and Sp3 are responsive to oxidative stress, modulate proteins important for vascular development, and therefore may play a significant, as of yet undiscovered, role in placental angiogenesis.

### 6.6. STAT-3

STAT-3 is part of a group of transcription factors that provide cellular regulation in response to cytokines and growth factors. This transcription factor has been shown to respond to reactive nitrogen species [126] and plays a central role in regulating a range of signaling molecules involved in cellular differentiation and proliferation in a variety of cell types [127, 128]. STAT-3 has also been linked to the attenuation of oxidative damage in different cellular compartments such as the mitochondrion [129]. Additionally, STAT-3 can respond to cytokine or oxidative signals by affecting the expression of angiogenic factors such as VEGF [117, 130]. The proangiogenic role of STAT-3 is linked to its ability to regulate both the expression and the secretion of MMP-2, MMP-9, and uPA, proteins involved in the modulation of trophoblast invasion [131].

Importantly this transcription factor also enhances the promoter activity of MMP-1 in the lung, as a consequence of exposure to cigarette smoke [90]. The activation of STAT-3 in response to cigarette smoke appears to be a robust response demonstrated in a variety of tissues including human bladder cells lines [132], human bronchial epithelial cell lines [133], and mouse brain endothelial cells where it has been associated with the regulation of antioxidant defense response [134]. Not only is the expression of STAT-3 important but so is the activation of STAT-3 which is regulated by the extent of STAT-3 phosphorylation. The amount of activated STAT-3 in isolated trophoblasts has been found to increase with maternal body mass index [135]. Aye and colleagues also demonstrated that STAT-3 phosphorylation can be regulated by the cytokine TNF-*α* [135], which is known to be elevated in obese individuals [136]. Furthermore, STAT-3 expression and activity are reduced in preeclampsia, which is associated with decreased invasiveness of trophoblasts [137]. Our own data along with that of others has demonstrated that altered trophoblast invasion is linked to insufficient spiral artery remodeling and decreased oxygenation of the placenta [6, 7]. Therefore STAT-3 may be an important regulator for the adaptive responses to oxidative stress within the placenta.

## 7. Conclusion

**Figure 3:**
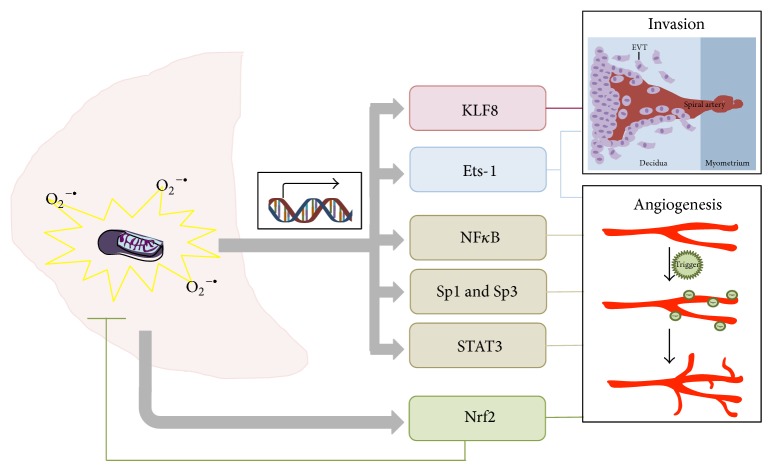
Placental oxidative stress triggers the expression of transcription factors to regulate angiogenesis and trophoblast invasion. Mitochondria within the placenta (depicted in pale pink) are a major producer of ROS, such as O_2_^−•^ which can cause a state of oxidative stress (illustrated in yellow). Oxidative stress within the placenta can act as a signaling pathway to influence the expression of transcription factors, such as KLF8, Ets-1, NF*κ*B, Sp1, Sp3, STAT-3, and Nrf2 (depicted by the grey arrows). These transcription factors regulate the expression and activity of proteins related to angiogenesis and trophoblast invasion (as shown by the lines linking to the invasion and angiogenesis panels).

We suggest that the transcription factors mentioned above warrant further investigation as to their roles in connecting increased oxidative stress with altered trophoblast invasion and placental angiogenesis (summarized in Figure 3). However, we do recognize that there remain many unexplored possibilities, which may provide further insights into mechanistic links between increased placental free radical production and changes to placental blood vessel development and function. One such example is cellular prion protein, thought to be involved in cellular copper regulation, which has been linked to altered placental oxidative stress and fetal growth and survival [138]. Elucidating the mechanisms that link adverse uterine conditions and adaptive placental development will lead to greater opportunities to develop therapeutic strategies for a number of obstetrical conditions which are associated with oxidative stress.
